# Epidemiological Clinical Features and Evolution of Gastroduodenal Ulcer Bleeding in a Tertiary Care Hospital in Spain, during the Last Seven Years

**DOI:** 10.1155/2013/584540

**Published:** 2013-12-09

**Authors:** Eugenia Lauret, Jesús Herrero, Lorena Blanco, Olegario Castaño, Maria Rodriguez, Isabel Pérez, Verónica Alvarez, Adolfo Suárez, Luis Rodrigo

**Affiliations:** Gastroenterology Service, University Hospital Central of Asturias, C/Celestino Villamil s/n, 33 006 Oviedo, Spain

## Abstract

*Background*. Gastroduodenal ulcer bleeding is a common medical emergency. The aim of this study was to analyze the characteristics of bleeding episodes and to identify changes in the clinical trends over seven years. *Methods*. Retrospective observational clinical study on a cohort of 272 consecutive adult patients with peptic ulcer bleeding, during the 2006–2012 period. *Results*. Mean annual admission rate was 12.8 per 100.000 inhabitants. Men were predominant (71%), with a mean age of 66.6 years. Comorbidities were present in 131 cases (48.2%) and 156 patients (57.4%) had received ulcerogenic drugs. Duodenal ulcer was the commonest location (61%). Endoscopic therapy was necessary in 183 cases (67.3%) and rebleeding occurred in 30 patients (11%). Overall mortality rate was 5.5%, with a significant association with the presence of comorbidities (*P* < 0.01). There were no differences in trends of annual hospitalization, clinical features at presentation, and outcomes during this 7-years period. *Conclusions*. Annual hospitalization rates and prognosis of peptic ulcer bleeding have remained unchanged in the study period. This may be due to the fact that the effect of improved approach on this condition is probably counteracted by risk factors such as older age, severe comorbidities, and ulcerogenic drugs consumption, which have also remained stable over recent years.

## 1. Introduction

Peptic ulcer bleeding remains a major clinical problem in the emergency setting, in Western countries. During the last decades, mortality rates from this gastrointestinal disorder have been reported to be stable, ranging between 5% and 15% worldwide [1, 2]. Despite increased knowledge about this condition, the use of improved diagnostics tools, the great advances in endoscopic therapy, and the routine use of ulcer treatment with proton pump inhibitors (PPIs) as well as eradication therapies against *Helicobacter pylori*, the published data on the changes in incidence and mortality for ulcer hemorrhage have been divergent [3–6].

In the nationwide population-based time-trend studies, a decline in general hospitalization rates was described over time, except among the elderly [4, 5]. By contrast, in regional population-based studies, the incidence of peptic ulcer bleeding was found unchanged [6]. The growing elderly population with presence of comorbidities and the gradual increase in prescription number of low-dose aspirin (ASA), nonsteroidal anti-inflammatory drugs (NSAID), and other antiplatelet or anticoagulant drugs have been recognized as some of the reasons for the expected changes on time trends and have been less significant than expected [2, 7, 8]. It is well documented that age is a main prognostic factor and that drugs that interfere with the defense mechanisms of the gastrointestinal mucosa and/or with the normal hemostasis may induce peptic ulcer bleeding [2, 9].

The aim of this study was to describe the clinical characteristics and endoscopic findings of peptic ulcer bleeding and the evolution of patients in a defined population from northern Spain during a 7-year period.

## 2. Material and Methods

The University Hospital Central of Asturias is a tertiary center covering a mean population of 303.163 inhabitants (aged 14 years or older). During the study period 2006–2012, the estimated population remained stable, with no significant annual variations. In total, 1255 episodes of nonvariceal upper gastrointestinal bleeding were attended in our hospital in the last seven years. Of these, 272 consecutives cases were hospitalized, with a diagnosis of bleeding peptic ulcer by early endoscopy. The exclusion critera were the presence of ulcer perforation, stomal localization or malignant ulceration. If the same patient had been hospitalized more than once for recurrent bleeding during the study period, only the first admission was considered for the analysis.

Data regarding age, gender, number and nature of comorbid illnesses, use of drugs, endoscopic findings according to Forrest classification [10], endoscopic treatment, and outcome by each bleeding episode were registered. Cardiovascular disease (defined by cardiac arrhythmias or ischemic heart disease), chronic liver disease, chronic obstructive pulmonary disease, chronic renal insufficiency, stroke, and simultaneous cancer of any localization were considered as comorbidities.

Mortality was defined as death occurring within the hospital admission up to 30 days and was classified as bleeding-related or nonbleeding-related death. Persistent bleeding was considered as failure to achieve hemostasis during the endoscopy. Rebleeding was defined as the presence of hematemesis melena, and/or recurrent hypotension and tachycardia, and/or decrease in blood hemoglobin >2 g/dL after successful endoscopic treatment with hemodynamic stability for at least 24 hours.

The design was a retrospective observational study. Despite being a noninterventional study, since the management of patients was performed according to standard clinical practice [11], all patients had signed informed consent for all diagnostic and therapeutic maneuvers required in each case. Endoscopic procedures were performed in the endoscopy unit by specialists Staff or by trained medical residents under supervision. Injection therapy and clips were the available endoscopic hemostatic techniques for peptic ulcer bleeding in our hospital. Epinephrine injection was used in combination with clips or sclerosant administration. Only these two latter modalities were used in a small number of cases as monotherapy in patients without actively bleeding ulcers. The hemostatic procedure was selected according to personal preference and expertise.

At entry, all patients received intravenous antisecretory therapy with proton pump inhibitors (except those with a known allergy to this drug), given as 80 mg bolus, followed by a continuous infusion of 8 mg per hour, during 3 days [12].

## 3. Statistics

Results are expressed as mean ± SD and as percentages. The *χ* ^2^ test was employed for qualitative variables, with Yates' correction for continuity and Fisher's exact test. Comparisons between quantitative variables were performed by Student's *t* test and analysis of variance. Logistic regression was used to identify independent risk factors for rebleeding or death. The total annual incidence rates were calculated by using the number of patients with bleeding peptic ulcer divided by the total population in each year. All statistical analyses were performed using SPSS statistical software version 15.0 (SPSS Inc., Chicago, IL). A *P* value of less than 0.05 was considered statistically significant.

## 4. Results

### 4.1. Clinical Characteristics of Patients

**Figure 1:**
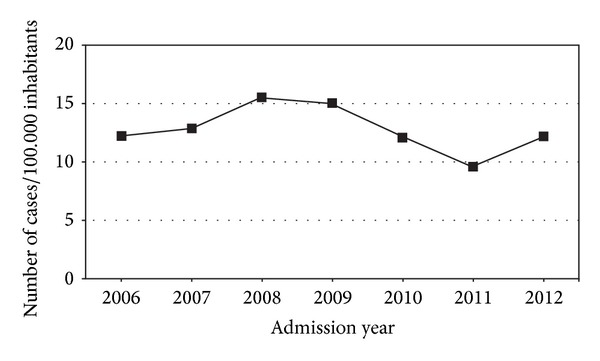
Annual hospitalization rates for peptic ulcer bleeding during seven years (2006–2012).

During the study period, 272 patients with bleeding peptic ulcer were admitted in our hospital. Of them, 251 (92.3%) remained at the gastroenterology department; 7 patients (2.6%) were initially hospitalized in the intensive care unit and the remaining 14 cases (5.1%) were attended in other departments. Mean annual admission rate (AAR) per 100.000 inhabitants was 12.82 and remained stable over the last seven years (Figure 1).

The mean age was 66.6 ± 17.3 years (range, 19–99) and men were predominant (193/272 [70.9%]; mean ratio M/F = 2.44). One hundred and thirty-one patients (48.2%) had associated at least one comorbid condition, and the most frequent was cardiovascular disease (90/131 [68.7%]) followed by chronic liver disease (15/131 [11.5%]). In relation to drugs consumed before admission, 55 patients (20.2%) were taking AAS, 43 (15.8%) other platelet aggregation inhibitors or anticoagulants, 32 (11.8%) NSAID, and 26 (9.6%) a combination of them. In 45 cases (16.5%) there was a history of prior peptic ulcer. There were no differences regarding epidemiological and clinical characteristics according to the year of admission (Table 1).

### 4.2. Endoscopic Findings

In total, 263 patients (96.7%) underwent early endoscopy (<24 hours) at entry. The location of peptic ulcer was duodenal in 165 patients (60.7%), gastric in 101 (37.1%), and simultaneous in the remaining 6 cases (2.2%). No statistically significant changes were found in the location of bleeding peptic ulcer over the years (*P* = 0.2). Fifty-five lesions (20.2%) were classified as Forrest I, 149 (54.8%) as Forrest II, and 68 (25%) as Forrest III. The frequency of endoscopic high-risk stigmata for rebleeding (ulcers classified as Forrest Ia, Ib, IIa, and IIb) was more similar in patients with duodenal ulcers than in those with gastric location (*P* = 0.2) (Table 2).

In total, 183 patients (67.3%) received endoscopic treatment (injection of epinephrine in combination with sclerosant or this latter in monotherapy) in 163 (89%), monotherapy with hemoclips in 2 (1.1%), and combined therapy with injection plus hemoclips in the remaining 18 (9.9%) cases. Endoscopic hemostasis was not achieved in 5 patients (2.7%). Only 15 of the 178 cases (8.4%) with apparent endoscopic hemostasis in the first procedure performed a second-look endoscopy.

### 4.3. Outcome after Bleeding Episode

Rebleeding after early endoscopy occurred in 30 patients (11%) with an average of 3.5 ± 4.9 days (range, 0–25). Of these, effective hemostasis was achieved in 24/27 cases (88.9%) with repeated endoscopy. The mean number of endoscopic procedures was 1.1 ± 0.4 explorations by case (range, 1–3). Eight patients (2.9%) required surgery for definitive treatment of bleeding.

Complication occurred in 15.1% (41/272) and death rate was 5.5% (15/272). The most common cause of complication was decompensation of underlying chronic disease in 37 patients. Endoscopic complications occurred in 4 patients (3 perforations and 1 aspiration pneumonia). Cause of death was mainly related to multiorgan failure, secondary to comorbidities in 7 cases and uncontrolled bleeding in the remaining 8 patients. There was no difference in mean age according to death cause (71.1 ± 16.7 versus 74.9 ± 14.8 years, resp., *P* = 0.7).

In the multivariate analysis, the risk of poor outcome was higher in patients with at least one comorbid condition and/or with lesions classified as high-risk stigmata for rebleeding (Table 3). There were no differences in annual rebleeding and death rates over the 7-year period.

Mean length of hospital stay was 6.5 days for all patients. After discharge, readmission for rebleeding occurred in 8 patients (2.9%) in median time of 4 days.

## 5. Discussion

Peptic ulcer bleeding is a common disorder that implies frequent hospitalizations and high use of resources. The study of characteristics and evolution of this disorder in our population had led to a better understanding of the current situation of this medical problem in our area and provides information on clinical practice. Patients living within hospital catchment area are in a very high percentage referred to our local hospital, so that the results obtained in this study are representative of this geographical location. Our results show a bleeding peptic ulcer incidence lower than the one reported by previous studies [2, 5, 6], with AAR per 100.000 inhabitants remaining stable, over the study period. This variability may be explained, in part, by the different clinical and demographic characteristics of the studied populations. Other possible reasons are due to changes in the prescription criteria of NSAID or PPIs use and different prevalence and eradication rates of *Helicobacter pylori* infection among distant geographical locations.

The clinical presentation of bleeding episodes does not differ from the data reported in other studies [1, 6, 7]. Patients were predominantly elderly men, with average ages in the seventh decade of life, and almost half of them had significant associated comorbidities. Besides, up to 57% of patients admitted to hospital for this disorder and have been taking drugs potentially harmful for the upper gastrointestinal tract.

In our study, patients with high-risk stigmata on index endoscopy had a greater chance of rebleeding. Although it has been described that a second-look endoscopy may be effective in these selected patients, this is not the current clinical practice in our experience, according to the available evidence [13], and this approach is reserved for patients at particularly increased risk of rebleeding considered on case by case basis (large ulcers or if the effectiveness of hemostasis at index endoscopy is questioned). On the other hand. We could estimate that about a fourth of cases might have been treated appropriately with a prompt discharge without a hospital admission [11, 14]. The reason is that some of these patients were admitted before an early endoscopy was performed and/or by decompensation of their underlying diseases due to bleeding episode. However, this data allowed us to identify a point improvement in the care of these patients, with the aim of decreasing the number of hospitalizations and associated costs in these low-risk cases.

The results of our study show a lower ulcer-related death rate than previously reported [1, 15], being comparable to the vigorous use of PPIs early at admission and during three days of perfusion than the registered in other European studies [3, 7], and confirm the slow tendency to reduce the mortality in this disease. Recent prospective studies have identified different predictors of mortality [1, 2, 15]. The two key interventions to reduce rebleeding and mortality from peptic ulcer, are to perform an early endoscopic treatment and vigorous use of intravenous PPI at admission [12, 16, 17], and both are used in a standardized manner with minimal variations between countries [11, 14]. Moreover, the decrease in surgical interventions rate has also caused a corresponding decrease in death rate. Therefore, the prognosis of the bleeding ulcer peptic did not seem to depend only on differences in hemorrhage management, but rather on the characteristics of the patients, and this could be related to the increasing age and comorbidities as described in other series. The presence of chronic associated diseases seems to be a very clinical important risk factor for mortality, more than previous positive history of peptic disease and the anatomical locations of lesions [1, 15].

The lessons of this study are based on data collection of a representative number of patients with peptic ulcer bleeding in the last seven years. Information obtained reflects the real situation of this problem in the population from our area who received their attention on “day-to-day conditions”, allowing the recognition of weaknesses and suggesting the need for implementation of improving strategies. The methodological limitations are given by the retrospective observational study design. A bias may be produced because our hospital is a referral center for other geographic areas that do not have specialized personnel and early endoscopy and therefore provides treatment to more serious situations. In this regard, to minimize this bias, this study only included cases of ulcer peptic bleeding in patients with permanent residence in the area of the hospital. Another criterion was the exclusion of episodes that had not performed endoscopy for diagnosis and in-hospital bleeding. These may include a subgroup of patients with greater severity or comorbidity, so their exclusion may influence the final results and prognosis, but in general the number of these cases is low. Error in coding or incomplete coding in admissions cannot be excluded, but there has not been any significant change in the recording process over our study period. No management criteria of peptic ulcer bleeding have substantially changed over the last seven years.

In conclusion, our study shows that the clinical features and prognosis of peptic ulcer bleeding have remained unchanged in the last years. This may be explained because patients are older, suffer more associated comorbidities, and/or take more drugs that promote bleeding, which contributes to reducing the effects of recent advances in medical and endoscopic management of this condition.

## Figures and Tables

**Table 1:** Demographic and clinical characteristics of patients with peptic ulcer bleeding.

	2006 (*n* = 37)	2007 (*n* = 39)	2008 (*n* = 47)	2009 (*n* = 46)	2010 (*n* = 37)	2011 (*n* = 29)	2012 (*n* = 37)	*P *
Male gender, *n* (%)	25 (67.6)	32 (82.1)	36 (76.6)	28 (60.9)	26 (70.3)	19 (65.5)	27 (73)	0.4
Age (mean ± SD, years) Range	69.7 ± 16.3 (20–93)	65.0 ± 18.62 (19–93)	63.7 ± 17.4 (21–91)	64.8 ± 18.2 (25–91)	68.3 ± 15.36 (28–88)	68.3 ± 18.0 (27–93)	67.9 ± 17.3 (28–99)	0.7
Comorbidities, *n* (%)	19 (51.4)	19 (48.7)	21 (44.7)	22 (47.8)	14 (37.8)	17 (58.6)	19 (51.4)	0.8
Drugs^a^, *n* (%)	20 (54.1)	20 (51.3)	27 (57.4)	27 (58.7)	23 (62.2)	14 (48.3)	25 (67.6)	0.7
Prior peptic ulcer, *n* (%)	5 (13.5)	3 (7.7)	9 (19.1)	6 (13.0)	11 (29.7)	5 (17.2)	6 (16.2)	0.3

^a^Aspirin (AAS), non-steroidal anti-inflammatory drugs (NSAID), antiplatelet therapy, and/or anticoagulant drugs.

**Table 2:** Differential clinical characteristics according to endoscopic findings.

	Gastric ulcer^a^ (*n* = 101)	Duodenal ulcer^a^ (*n* = 165)	*P *
Male gender, *n* (%)	63 (62.4)	127 (77)	<0.05
Age (mean ± SD, years) Range	68.5 ± 16.5 (26–97)	65.4 ± 17.9 (19–99)	0.1
Comorbidities, *n* (%)	48 (47.5)	80 (48.5)	0.9
Drugs^b^, *n* (%)	69 (68.3)	82 (49.7)	<0.01
Prior peptic ulcer, *n* (%)	14 (13.9)	30 (18.2)	0.4
Forrest classification, *n* (%)			
Active bleeding (Ia, Ib)	10 (9.9)	45 (27.3)	<0.001
Visible vessel (IIa)	28 (27.7)	29 (17.6)	
Adherent clot (IIb)	17 (16.8)	29 (17.6)	
Flat/Black spot (IIc)	20 (19.8)	22 (13.3)	<0.05^c^
White base (III)	26 (25.7)	40 (24.2)	0.9
Endoscopic treatment, *n* (%)	64 (63.4)	115 (69.7)	0.3

^a^Patients with gastric and duodenal ulcers were not considered.

^b^Aspirin, non-steroidal anti-inflammatory drugs (NSAID), antiplatelet therapy, and/or anticoagulant drugs.

^c^All ulcers were classified as Forrest II.

**Table 3:** Outcomes of patients with bleeding episode.

	Rebleeding (*n* = 30)	*P *	Death (*n* = 15)	*P *
Male gender, *n* (%)	23 (76.7)	0.5	13 (86.7)	0.2
Age (mean ± SD, years) Range	65.0 ± 14.9 (39–90)	0.5	73.4 ± 15.2 (50–99)	0.1
Comorbidities, *n* (%)	17 (56.7)	0.3	14 (93.3)	<0.01
Drugs^a^, *n* (%)	16 (53.3)	0.7	6 (40)	0.2
Prior peptic ulcer, *n* (%)	5 (16.7)	0.9	1 (6.7)	0.5
Duodenal ulcer, *n* (%)	19 (63.3%)	0.9	9 (60%)	0.9
Forrest classification, *n* (%)				
High-stigmata signs for rebleeding^b^	25 (83.3)	<0.01	10 (66.7)	0.6
Death, *n* (%)	4 (13.3)	0.07	—	—
Rebleeding, *n* (%)	—	—	4 (26.7)	0.07

^a^Aspirin, non-steroidal anti-inflammatory drugs (NSAID), antiplatelet therapy and/or anticoagulant drugs.

^b^Patients with gastric and duodenal ulcers were not considered. High-stigmata signs for rebleeding included ulcers classified as Forrest Ia, Ib, IIa and IIb.
